# CHR729 Is a CHD3 Protein That Controls Seedling Development in Rice

**DOI:** 10.1371/journal.pone.0138934

**Published:** 2015-09-23

**Authors:** Xiaoding Ma, Jian Ma, Honghong Zhai, Peiyong Xin, Jinfang Chu, Yongli Qiao, Longzhi Han

**Affiliations:** 1 National Key Facility for Crop Gene Resources and Genetic Improvement, Institute of Crop Science, Chinese Academy of Agricultural Sciences, Beijing, China; 2 State Key Laboratory of Cotton Biology, Institute of Cotton Research, Chinese Academy of Agricultural Sciences, Anyang, China; 3 Key Laboratory of Biology and Genetic Improvement of Horticultural Crops (North China), Ministry of Agriculture, Beijing Vegetable Research Center, Beijing Academy of Agriculture and Forestry Sciences, Beijing, China; 4 National Center for Plant Gene Research (Beijing), Institute of Genetics and Developmental Biology, Chinese Academy of Sciences, Beijing, China; University of Western Sydney, AUSTRALIA

## Abstract

CHD3 is one of the chromatin-remodeling factors that contribute to controlling the expression of genes associated with plant development. Loss-of-function mutants display morphological and growth defects. However, the molecular mechanisms underlying CHD3 regulation of plant development remain unclear. In this study, a rice CHD3 protein, CHR729, was identified. The corresponding mutant line (*t483*) exhibited late seed germination, low germination rate, dwarfism, low tiller number, root growth inhibition, adaxial albino leaves, and short and narrow leaves. *CHR729* encoded a nuclear protein and was expressed in almost all organs. RNA-sequencing analysis showed that several plant hormone-related genes were up- or down-regulated in *t483* compared to wild type. In particular, expression of the gibberellin synthetase gibberellin 20 oxidase 4 gene was elevated in the mutant. Endogenous gibberellin assays demonstrated that the content of bioactive GA_3_ was reduced in *t483* compared to wild type. Moreover, the seedling dwarfism, late seed germination, and short root length phenotypes of *t483* were partially rescued by treatment with exogenous GA_3_. These results suggest that the rice CHD3 protein CHR729 plays an important role in many aspects of seedling development and controls this development via the gibberellin pathway.

## Introduction

Chromatin is a macromolecular complex that consists of DNA, protein, and RNA. At least three processes control chromatin assembly and regulation: DNA methylation, histone modification, and ATP-dependent chromatin remodeling [1]. Chromatin is highly dynamic and undergoes structural alterations in response to developmental changes and changes in the cellular environment [2, 3]. Dynamic regulation of chromatin structure is important for transcriptional regulation and other functions in higher eukaryotes, and many coordinating control factors are needed. One such factor is chromatin-remodeling factor, a member of the CHD family, which plays a substantial role in cellular development and chromatin-mediated transcriptional regulation [4, 5].

CHD proteins contain *c*hromo, *h*elicase/ATPase, and *D*NA-binding domains, and belong to the *s*ucrose *n*on-*f*ermenting (SNF2)-like family of ATPases. CHD proteins in higher eukaryotes fall into three subfamilies [6]. One of these, the CHD3 subfamily, is widely distributed in animals and plants and has been intensely studied. Early studies found that CHD3 members served as repression factors in various types of developmental regulation [7]. However, more recent studies indicated that CHD3 members were also involved in gene activation [8–11]. One example is the CHD3 protein PICKLE (PKL) in Arabidopsis. PKL was first found to act as a repressor in cellular differentiation, embryonic transition, and meristem development in leaf and carpel tissues [12–14], but more recent research found that the expression levels of root meristem genes decreased in a *pkl* mutant, and that root meristem activity was controlled by PKL [8].

In general, CHD3 proteins regulate gene expression in plants by promoting trimethylation of lysine 27 of histone H3 (H3K27me3). In the Arabidopsis *pkl* mutant, H3K27me3 levels were substantially lowered in H3K27me3-enriched genes, indicating a role for *PKL* in the modification of repressed chromatin [15]. Chromatin immunoprecipitation experiments showed that PKL directly interacted with the promoter region of H3K27me3-enriched genes. For example, during germination, PKL protein acted directly on the promoters of *LEAFY COTYLEDON1* and *LEAFY COTYLEDON2*. As a consequence, the expression of embryonic identity-related genes was repressed by PKL and seed germination was promoted [16]. CHD3 protein can also interact with dimethylated histone lysine 4 (H3K4me2). CHD3 proteins in rice also interact with H3K27me3 and H3K4me2 to control target genes during development [17].

Although a number of CHD3 subfamily proteins were described in previous studies, the mechanisms underlying their mediation of gene regulation are not yet fully understood. This is particularly the case for the relationships between target genes and developmental processes in different tissues. To examine these processes further, it is necessary to identify more CHD3 genes, screen downstream genes, and analyze relationships between target genes and phenotypic variation. This will not only help to elucidate the mechanisms underlying CHD3 proteins and gene regulation, but will also uncover the relationships between dynamic regulation of chromatin structure and developmental processes.

This study examined rice mutant *t483*, which displayed a number of characteristic morphological and growth features such as defective seed germination, dwarfism, low tiller number, root growth inhibition, and short and narrow leaves. Map-based cloning was used to isolate the gene responsible for the *t483* phenotypes. The defect was caused by mutation in the gene encoding the CHD3 protein CHR729 (*CH*D-*R*elated). RNA sequencing showed that expression of the gene encoding an active gibberellin synthetase, gibberellin 20 oxidase 4, was elevated in the mutant. Endogenous gibberellin analysis revealed that the content of bioactive GA_3_ was reduced in the *t483* mutant compared with wild type (WT). The *t483* mutant was responsive to gibberellin, with exogenous GA_3_ treatment partially correcting defective root/shoot development and seed germination. This suggested that *CHR729* exercised control of development through the gibberellin pathway.

## Materials and Methods

### Plant materials and growth conditions

The *t483* mutant (*japonica* cv. Nipponbare) was obtained from an EMS (Ethyl methanesulfonate)-mutagenized population. Nipponbare was used as a WT line for phenotypic observation and gene expression analysis. All materials for crossing and analysis were grown in the experimental field at the Chinese Academy of Agricultural Sciences, Beijing and Sanya.

For germination analysis, rice seeds were soaked in tap-water at 37°C in the dark for three days. The soaked seeds were incubated at 28°C with 12 h of light and 12 h of darkness for eight days, then seed germination rate was measured by counting only those seeds with shoots longer than 2 cm. For gibberellin treatment, mutant and WT seeds were surface sterilized in 2.5% NaClO, soaked with tap-water in sterile Petri dishes at 37°C in the dark for one day. Seeds were then soaked in different concentrations of gibberellin acid solution and incubated at 28°C with 12 h of light and 12 h of darkness.

### Chlorophyll content measurement and transmission electron microscopy analysis

Chlorophyll contents were measured using a spectrophotometer according to the method of Arnon [18] with minor modifications. Briefly, equal weights of freshly collected second top leaves from two-week-old seedlings were marinated in 95% ethanol for 48 h in darkness. For thorough chlorophyll extraction tubes were periodically inverted five times. Residual plant debris was removed by centrifugation. The supernatants were used to measure chlorophyll content by a DU 800 UV/Vis spectrophotometer (Beckman Coulter) at 665, 649 and 470 nm.

For transmission electron microscopy analysis, leaf samples from two-week-old plants growing in paddy field were first fixed in 2% glutaraldehyde solution and then transferred into 1% OsO_4_ for two days. After fixation, samples were stained with uranyl acetate and dehydrated in an ethanol series, and then embedded in Spurr’s medium before ultrathin sectioning. Samples were stained with uranyl acetate again and observed with a transmission electron microscope (Hitachi H-7650, Japan).

### Map-based cloning of the mutated gene in *t483*

To map the mutated gene, *t483* was crossed with cv. 93–11 (*indica*). The plants with the mutant phenotype in F_2_ population were selected for a genetic linkage analysis. Molecular markers distributed throughout the rice genome were chosen for preliminary mapping [19, 20]. Additional *In*sertion-deletion (IN) markers for fine mapping were developed according to the DNA sequence difference between *japonica* and *indica*. PCR procedure was as following: 95°C for 5 min, followed by 35 cycles of 95°C for 30 s, annealing for 30 s, extension 72°C for 30 s, and a final extension at 72°C for 5 min.

### Generation of transgenic rice plants

Because the genomic sequence of *CHR729* was very large, containing 13,848 bp (not including promoter sequence), it was difficult to construct a complementary transformation plasmid. We therefore used a *pCUbi1390-ΔFAD*_*2*_ RNAi vector to generate a *CHR729-*RNAi construct [21]. The specific region of *CHR729* used for the RNAi construct was identified by alignment with the basic local alignment search tool (http://www.gramene.org). A 346 bp specific fragment of the *CHR729* gene was amplified with primer pairs RNAi-SF/ RNAi-SR and RNAi-AF/ RNAi-AF, then cloned into the *pCUbi1390-ΔFAD*_*2*_ vector as described by Mao et al. [22]. The RNAi construct was introduced into wild type Nipponbare by *Agrobacterium tumefaciens*-mediated transformation as previous report and empty *pCUbi1390* vector was also introduced as a control [23].

### Quantitative real-time PCR analysis

Total RNA was extracted from various tissues using RNA Prep Pure Plant Kit (Tiangen Co., Beijing), and was reverse transcribed using a SuperScript II Kit (TaKaRa), according to the user’s manual. qRT-PCR (quantitative real-time PCR) analyses were performed using the 7900 HT Fast Real-Time PCR System (ABI). *UBIQUITIN* gene (*Os03g0234200*) was chosen as a reference gene. Reactions containing SYBR premix (TaKaRa) were carried out in final volumes of 20 μL with 2 pmol of the appropriate primers. The 2^—ΔΔCT^ method was used to calculate relative levels of gene expression [24].

### Subcellular localization of CHR729

To determine the subcellular localization, the *CHR729* cDNA fragment was amplified (primer pair GFP-F/GFP-R) and ligated into *pA7-GFP* vector (*p35S-CHR729-GFP*). As a control, the cDNA of a previously characterized nuclear protein, OsMADS3, was fused to the *mCherry* gene (*p35S-OsMADS3-mCherry*) [25]. Protoplasts were isolated from rice seedlings, and co-transfected with the *p35S-CHR729-GFP* and *p35S-OsMADS3-mCherry* vectors. The transformed protoplasts were incubated at 28°C in darkness for 16 h before detection. Fluorescence of GFP was observed using a confocal laser scanning microscope (Leica TCS SP5).

### RNA-sequencing

Two-week-old whole WT and *t483* seedlings were immediately frozen in liquid nitrogen and stored at -80°C. Material from five different plots was pooled together. Total RNA was extracted using a RNA Prep Pure Plant Kit (Tiangen Co., Beijing), and treated with RNase-free DNase I (NEB, Ipswich, MA, USA) to remove any genomic DNA contamination. For each sample, at least 10 μg of total RNA was used for illumina HiSeq2000 sequencing conducted at Beijing Novo Co. (Beijing). After sequencing, the raw reads were first purified by trimming adapter sequences and removing low-quality sequencing date. The clean reads were mapped to the reference genome of *japonica* cv. Nipponbare using SOAP2 software [26]. Genes differentially expressed (DEGs) between *t483* and WT were identified using DEGseq R package (1.12.0; TNLIST, Beijing). P-values were adjusted using the Benjamini and Hochberg method [27]. Corrected p-values of 0.001 and log2 (fold change) of ±1 were set as the threshold to determine significant differential expression.

Gene ontology (GO) analysis was performed using the open-source MAS3 database (http://bioinfo.capitalbio.com/mas3/). A threshold of a two-fold change in gene expression levels and a FDR of <0.05 were used to identify DEGs. The p-values and FDRs of DEGs were calculated as previously reported [28].

### Determination of endogenous GAs levels

500 mg of the plant material powder was extracted with 5 mL of 90% aqueous methanol (MeOH). Simultaneously 2 ng of each D-labelled GA compound was added to the extracting solvents as internal standards for GAs content measurement. MAX and MCX Cartridges (6 mL, 500 mg) were purchased from Waters Corporation (Milford, MA, USA). The MAX cartridge was activated and equilibrated with 10 mL MeOH, water, 5% NH4OH, 90% MeOH in turn, while MCX with 10 mL MeOH, water and 90% MeOH. After the two columns were connected with an adapter, the crude extracts were subjected to the tandem cartridges. Then the MAX cartridge was disconnected and washed with 5% NH_4_OH in 5% MeOH, MeOH in sequence. At last GAs were eluted with 2% FA in 90% MeOH. After dried with N_2_ stream, the eluent was reconstructed with 200 μL 80% MeOH and subjected to UPLC-MS/MS analysis. GAs analysis was performed on a quadrupole linear ion trap hybrid mass spectrometer (QTRAP 5500, AB SCIEX, Foster City, CA) equipped with an electrospray ionization source coupled with a UPLC (Waters, Milford, MA, USA). Five microliters of each sample were injected onto a BEH C18 column (100 mm*2.1 mm, 1.7 μm). The inlet method was set as follows: mobile phase A: 0.05% acetic acid in water, B: acetonitrile. Gradient: 0–17 min, 3% B to 65% B; 17–18.5 min, 65% B to 90% B; 18.5–19.5min, 90% B; 19.5–21 min, 90% B to 3% B; 21–22.5 min, 3% B. GAs were detected in negative multiple reaction monitoring (MRM) mode. Each GA compound was quantified with a MRM transition and qualified with another one. The source parameters were set as: IS voltage -4500 V, TEM 600°C, GS1 45, GS2 55 and curtain gas 28.

## Results

### Phenotypic characterization of the rice *t483* mutant

**Fig 1:**
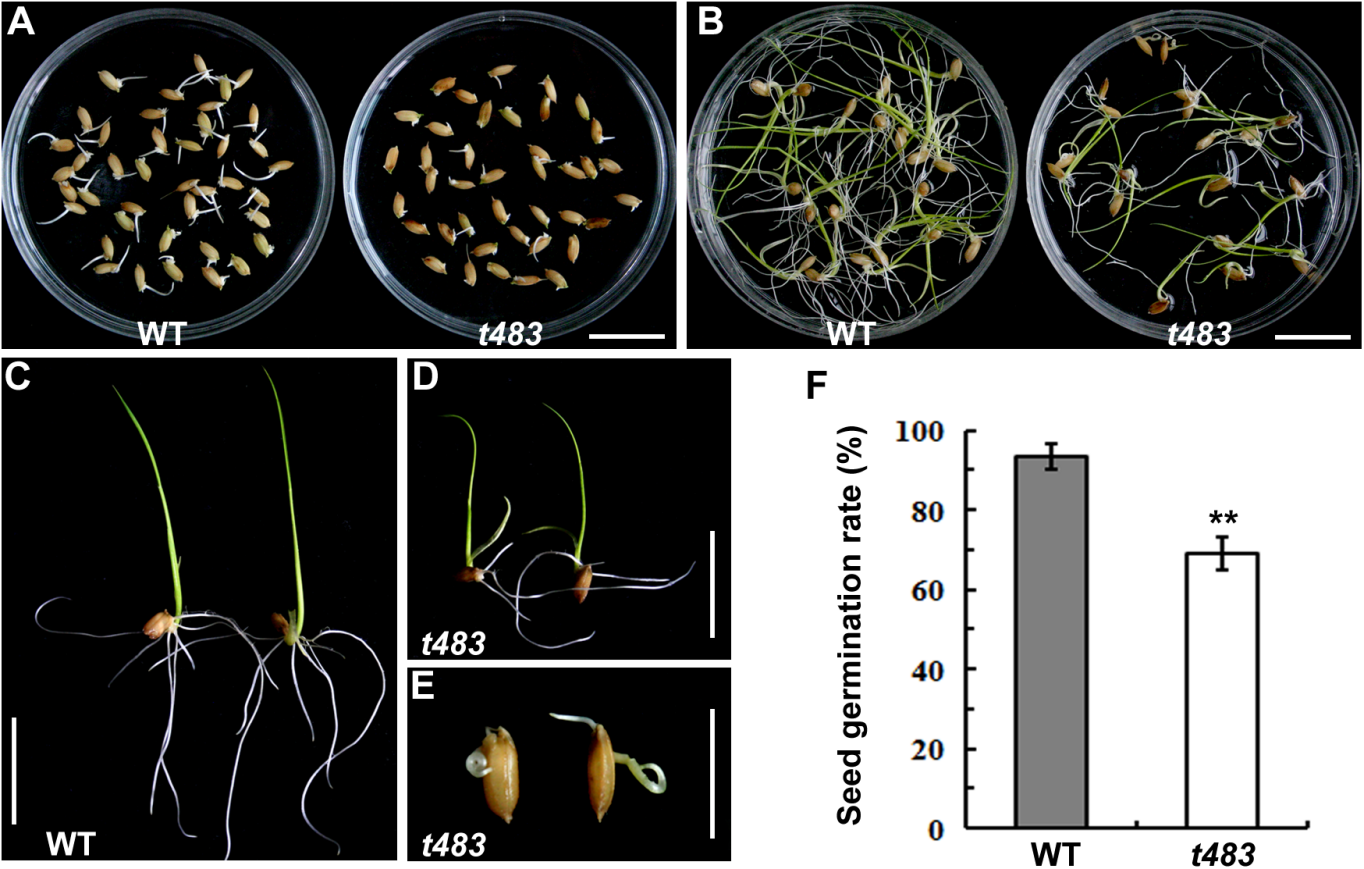
Seed germination of WT and *t483*. A, Germination of WT and *t483* seeds after 3 days. B–E, Seedlings of WT and *t483* at 8 days post-germination. F, Seed germination rate. Values are means ±SD of three independent experiments. Significance of differences between WT and *t483* was determined by Student’s *t-*test (**P<0.01). Scale bars: 2 cm (A–D); 1 cm (E).

When compared to WT rice plants, *t483* plants exhibited abnormal growth at every stage of development. The germination rate was significantly lower in the *t483* mutant than in WT, with a clear difference first becoming apparent three days after germination (Fig 1A). Eight days post-germination, shoots of *t483* were shorter than those of WT and roots in the mutant were shorter and lower in number (Fig 1B–1D). Furthermore, approximately 30% of the *t483* mutant seeds arrested at the germination stage (Fig 1B, 1E and 1F). These results indicate that *t483* is defective in seed germination.

**Fig 2:**
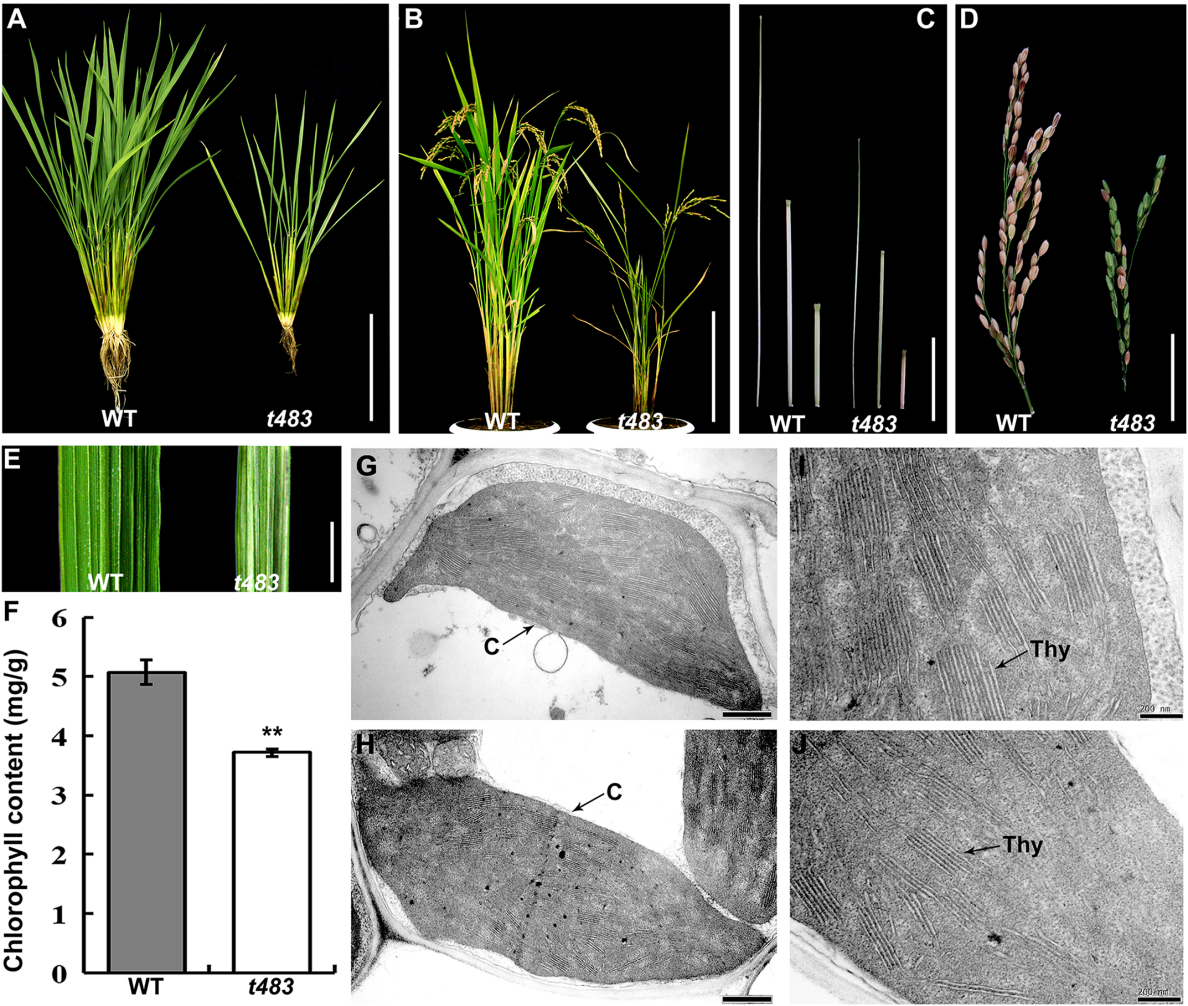
Phenotypic characteristics of WT and *t483*. A, Plants at the tillering stage after removal of soil. B, Mature plant stage. C, Three uppermost internodes from the main tiller. D, Panicles of WT and *t483*. E, Adaxial side of leaf segments. F, Leaf chlorophyll contents in WT and *t483*. Values are means ±SD (n = 3) (**P<0.01). G–H, Ultrastructure of chloroplasts in adaxial mesophyll cells of WT (G) and *t483* (H). I–J, Thylakoid lamellar structure of WT (I) and *t483* (J). *C* chloroplast, *Thy* thylakoid lamellar. Scale bars: 10 cm (A, C); 25 cm (B); 5 cm (D); 1cm (E); 0.5 μm (G, H); 0.2 μm (I, J).

The *t483* mutant was also defective at other development stages. At the tillering stage, stature and tiller numbers were reduced compared to WT (Fig 2A). At the mature stage, mutant plants were reduced in height and had smaller and fewer panicles than WT plants (Fig 2B–2D, Table 1). In addition, leaf blade lengths and widths were reduced in the *t483* mutant compared to WT. Mutant leaves exhibited pale coloration on the adaxial side (Fig 2E, Table 1) and normal coloration on the abaxial side (data not shown). Spectrophotometric analysis showed that chlorophyll levels were 27% lower in *t483* than in WT (Fig 2F). Consistent with the alteration in leaf color, the numbers of thylakoid lamellar and stacked grana in chloroplasts of adaxial mesophyll cells were lower in *t483* than in WT, as determined using transmission electron microscopy (Fig 2G–2J). The chloroplast structure and thylakoid lamellar were, however, developed normally in abaxial mesophyll cells of the mutant (data not shown). In summary, the *t483* mutant had morphological and growth defects that affected seed germination, plant height, tillering, panicle, leaf color, and leaf and root dimensions.

**Table 1:** Phenotypic comparisons of the *t483* mutant and wild-type Nipponbare (Beijing).

Genotype	Plant height (cm)	Panicles no.	Panicle length (cm)	Flag leaf length (cm)	Flag leaf width (cm)
WT	88.7±1.5	25.3±2.5	21.1±1.4	29.9±2.2	1.8±0.06
*t483*	56.3±2.5	10.2±2.1	14.0±1.2	19.3±1.3	0.67±0.05
*P*-value	1.7×10^−4^	0	0.002	0.007	1.9×10^−5^

Data are presented as means ± SE. Flag leaf widths were measured through the middle region of leaves at the mature stage. Significance of differences between WT and *t483* was detected using Student’s *t*-test (n = 20).

### Map-based cloning of the mutated gene in *t483*

**Fig 3:**
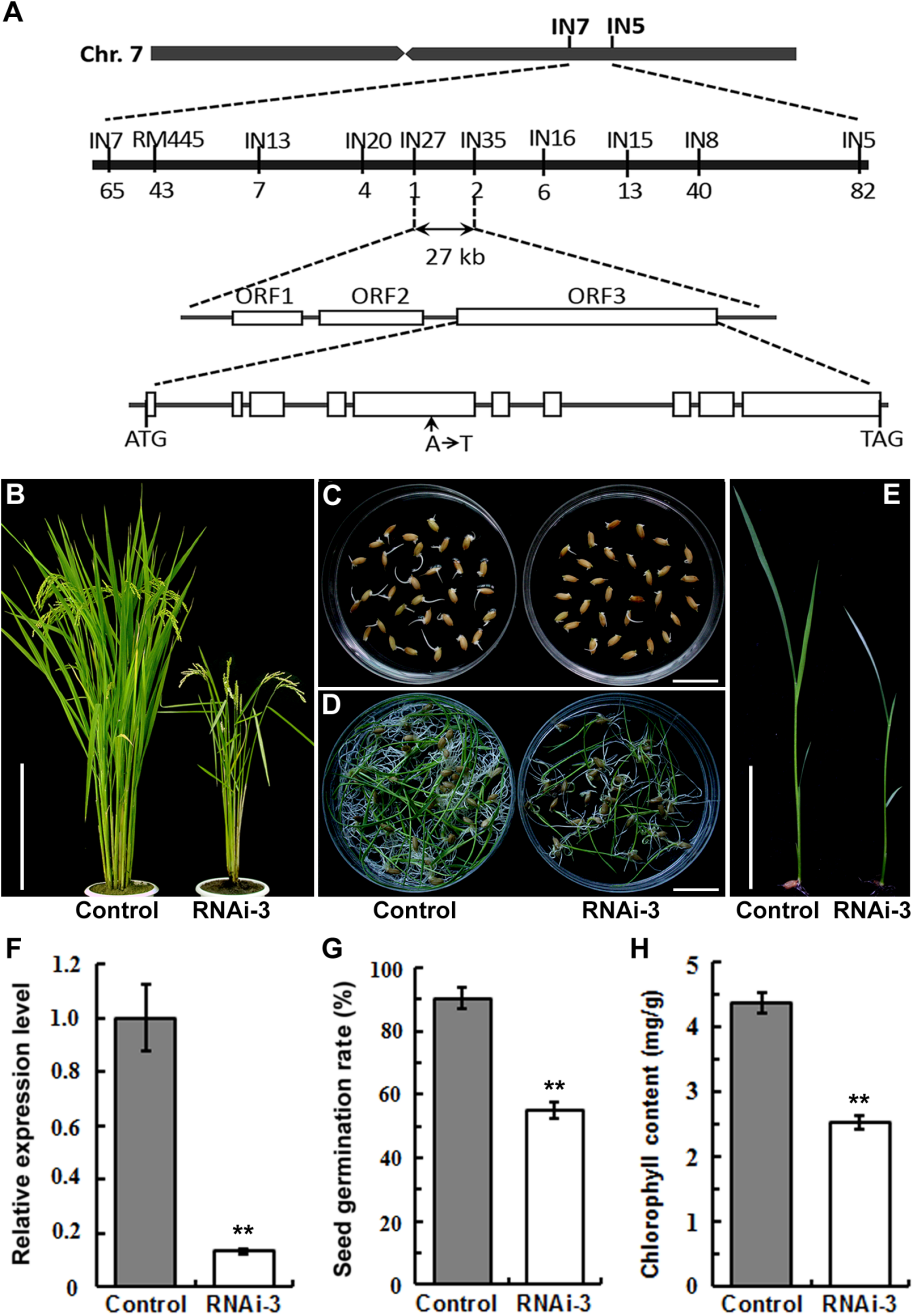
Map-based cloning and confirmation of the *CHR729* gene. A, Map of the genomic region containing the *t483* mutant locus of interest. Numerals below the corresponding markers indicate the number of recombinants identified among F_2_ plants with the mutant phenotype mutant. The mutated gene was located in a 27 kb region between markers IN27 and IN35. Three ORFs were predicted in the mapped region. Sequencing analysis revealed that an A to T substitution in the fifth exon of the ORF3 resulted in a stop codon in *t483*. B, Phenotypes of control and typical T_2_ transgenic knockdown plants (RNAi-3) at the heading stage. C, Germination of control and RNAi-3 seeds at 3 day. D, Control and RNAi-3 seedlings 8 days post-germination. E, Two-week-old seedlings. F, Expression analysis of *CHR729* in leaves of control and RNAi-3 by qRT-PCR. G, Seed germination rates in control and RNAi-3 plants. H, Chlorophyll contents in control and RNAi-3 plants. Chlorophyll was extracted from above-ground parts of plants shown in (E). Values are means ±SD (n = 3, **P<0.01). Scale bars: 25 cm (B); 2 cm (C, D); 5 cm (E).

Segregation analysis indicated that the *t483* phenotype was controlled by a single recessive gene (55:174, χ^2^_1:3_ = 0.118, *P*>0.70). The mutated gene was isolated by positional cloning. An F_2_ population with 1,350 recessive mutant plants was developed from a cross between *t483* and *indica* cv. 93–11. The mutated gene was located in a 27 kb region between markers IN27 and IN35 (Fig 3A). Three predicted open reading frames (ORFs) were predicted in this region, and the genomic sequences of each were obtained from WT and *t483*. A single nucleotide substitution of T for A in ORF3 in *t483* was predicted to encode an early stop codon (Fig 3A). This nucleotide change was confirmed in the cDNA sequence.

Just after we analyzed the mutated gene in *t483*, two similar mutants, named *chr729* and *oschr4*, were reported elsewhere [17, 29]. The location and sequence of these mutants suggested that *CHR729/OsCHR4* was a candidate for *t483*. To investigate this further, we generated several transgenic lines by introducing the specific RNA interference construct into Nipponbare plants. In a typical homozygous T_2_ line, RNAi-3, *CHR729* transcript levels were significantly lower than in control, as determined by real-time PCR analysis (Fig 3F). Phenotypic evaluation of RNAi-3 revealed that plant height (Fig 3B), seed germination (Fig 3C, 3D and 3G), chlorophyll content (Fig 3E and 3H), panicle number and length, and the length and width of the flag leaf were also significantly reduced in the RNAi-3 line compared to control (Table 2). The phenotype of the silenced plants mimicked that of the *t483* mutant. Together, the sequence and phenotype data suggested that the defect in the *t483* line was caused by a mutation in *CHR729/OsCHR4*. Henceforth, *t483* is used to denote the rice mutant line and *CHR729* is used for the gene name.

**Table 2:** Phenotypic comparisons of control and homozygous T_2_ transgenic plants of RNAi-3 line (Sanya).

Genotype	Plant height (cm)	Panicles no.	Panicle length (cm)	Flag leaf length (cm)	Flag leaf width (cm)
Control	82.1±2.1	19.7±2.1	19.4±1.0	22.3±1.9	1.7±0.3
RNAi-3	50.7±3.1	9.1±1.1	11.3±1.6	14.4±1.2	0.61±0.03
*P*-value	0.0003	0.005	0.0031	0.0064	1.8×10^−6^

Data are presented as means ± SE. Significance of differences between control and RNAi-3 was detected using Student’s *t*-test (n = 5).

Sequence comparison between genomic DNA and cDNA indicated that *CHR729* contained ten exons and nine introns, and encoded a 2259 amino acid protein. CHR729 belonged to the CHD3 protein subfamily and contained a PHD zinc finger domain, two chromo domains, a SNF2-related helicase domain, and a DNA-binding domain.

### *CHR729* is ubiquitously expressed and encodes a nuclear protein

**Fig 4:**
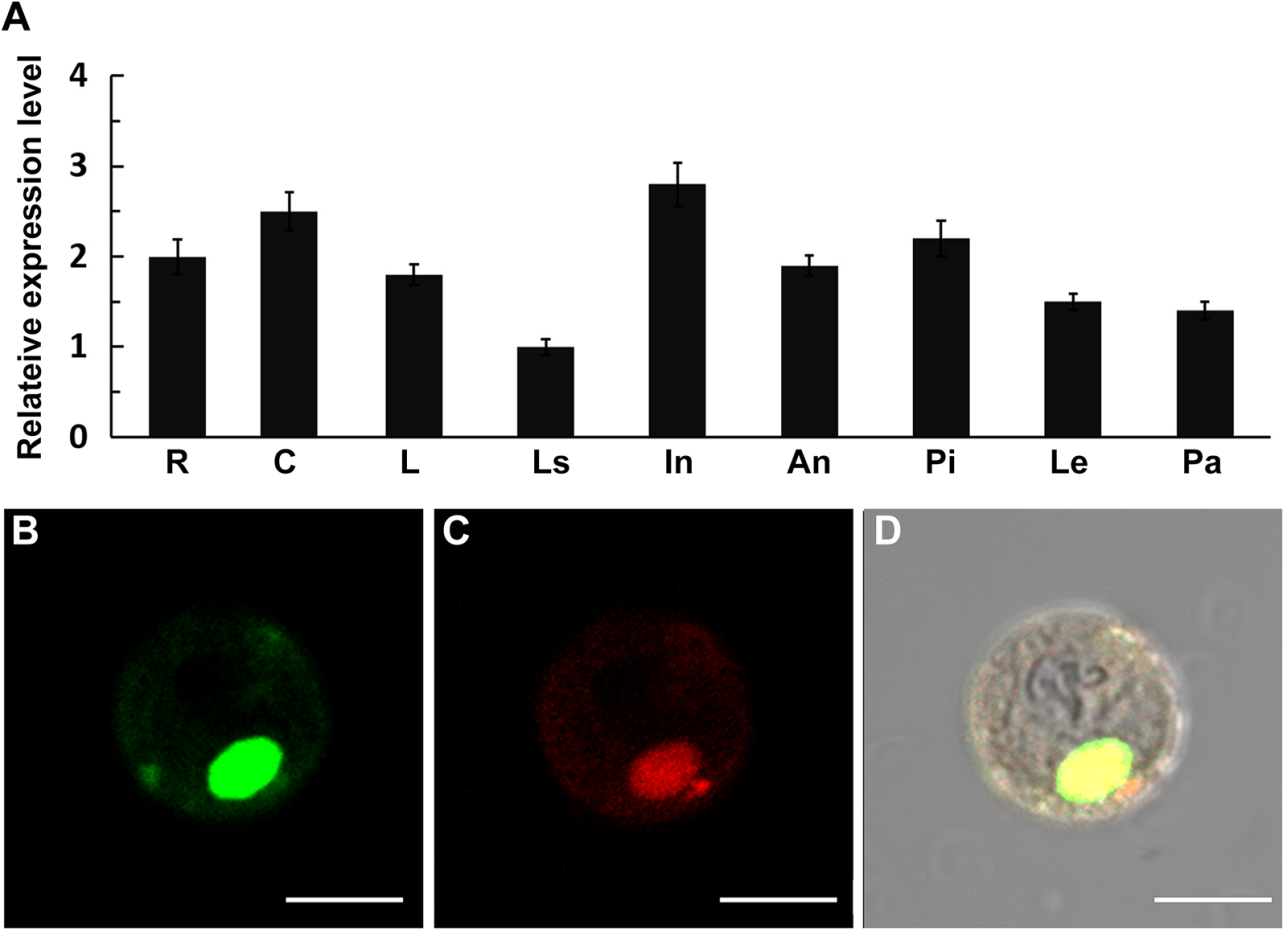
Expression analysis of *CHR729* and subcellular localization of the encoding protein. A, qRT-PCR analysis of *CHR729* expression in WT roots (R), culms (C), leaves (L), leaf sheaths (Ls), and young 3 cm inflorescence (In). Dissected anthers (An), pistils (Pi), lemmas (Le) and paleas (Pa) at inflorescence stage 9 were also analyzed. Values are means ±SD of three replicates. B–D, *CaMV35S*:CHR729-GFP fusion protein localization in rice protoplast. B, Subcellular localization of CHR729-GFP fusion protein. C, Subcellular localization of MADS3-mCherry fusion protein (nuclear marker). D, Merged image of (B) and (C) in bright field. Scale bars: 10 μm (B–D).

To examine the spatial expression pattern of *CHR729*, qRT-PCR analysis of endogenous *CHR729* transcripts was performed using total RNA isolated from different WT tissues. As shown in Fig 4A, *CHR729* was expressed in all the examined organs, including culms, leaf blades and sheaths, young inflorescences, and seedling-stage roots. *CHR729* was also expressed in flower organs, including anthers, pistils, lemmas, and paleas. *CHR729* therefore appeared to be constitutively expressed, which was consistent with the morphological defects observed in *t483* at many developmental stages.

To determine the subcellular localization of CHR729, a GFP fusion construct was transiently expressed in rice leaf protoplasts. The resulting GFP signal co-localized with the nuclear marker OsMADS3-mCherry [30] (Fig 4B–4D), suggesting that CHR729 was located in the nucleus.

### Expression of genes putatively related to phytohormone function is altered in the *t483* mutant

**Fig 5:**
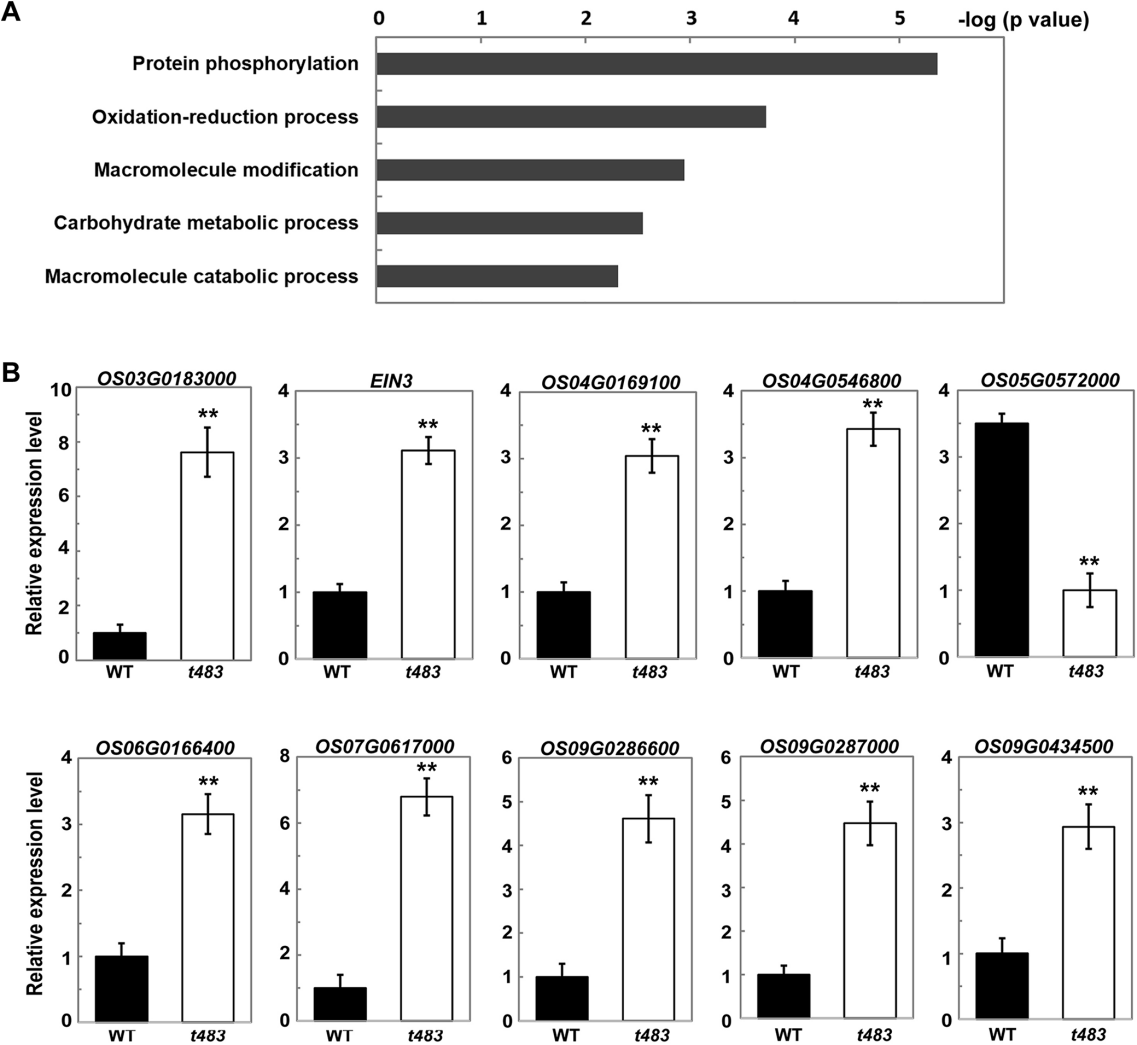
Analysis of differentially expressed genes in young *t483* seedlings. A, Identification of GO biological process categories for genes differentially expressed in *t483*. Negative logarithms (base 10) of the adjusted P values were used as the bar lengths. B, qRT-PCR analysis of expression in ethylene signaling-related genes. Values are means ±SD of three replicates. Student's *t-*test was used to determine significant differences in expression (**P<0.01).

To understand the molecular mechanism of *CHR729* in the regulation of plant development, gene expression changes were analyzed in young *t483* seedlings using RNA sequencing (RNA-seq). Transcriptional analysis showed that 346 and 154 genes were up- and down-regulated, respectively, in *t483* relative to WT (P<0.05; S1 Table). Many of the differentially expressed genes (DEGs) were identified as transcription factor genes (S2 Table). Gene ontology (GO) enrichment analysis found that five GO categories were enriched in the group of DEGs (Fig 5A, S3 Table). The most enriched category was “protein phosphorylation”, and this group included genes encoding protein kinases. Of the DEGs, several were predicted to involve in phytohormones such as auxin, abscisic acid (ABA), cytokinins, gibberellin, and ethylene (S4 Table). Only three of the total 19 phytohormone genes were downregulated, and more than half were involved in ethylene signaling. The RNA-seq results were confirmed using qRT-PCR (Fig 5B). One of the upregulated genes, *EIN3* (*Ethylene-insensitive 3*), encodes a protein that, after activation by EIN2, activates downstream transcription factors to induce various ethylene responses [31–34]. In addition to *EIN3*, expression of several other genes encoding ethylene-responsive transcription factors was elevated in *t483* compared to WT (S4 Table, Fig 5B). These results suggest that altered phytohormone signaling might contribute to the morphological and growth defects observed in the *t483* line.

### Responses of the *t483* mutant to gibberellins

**Fig 6:**
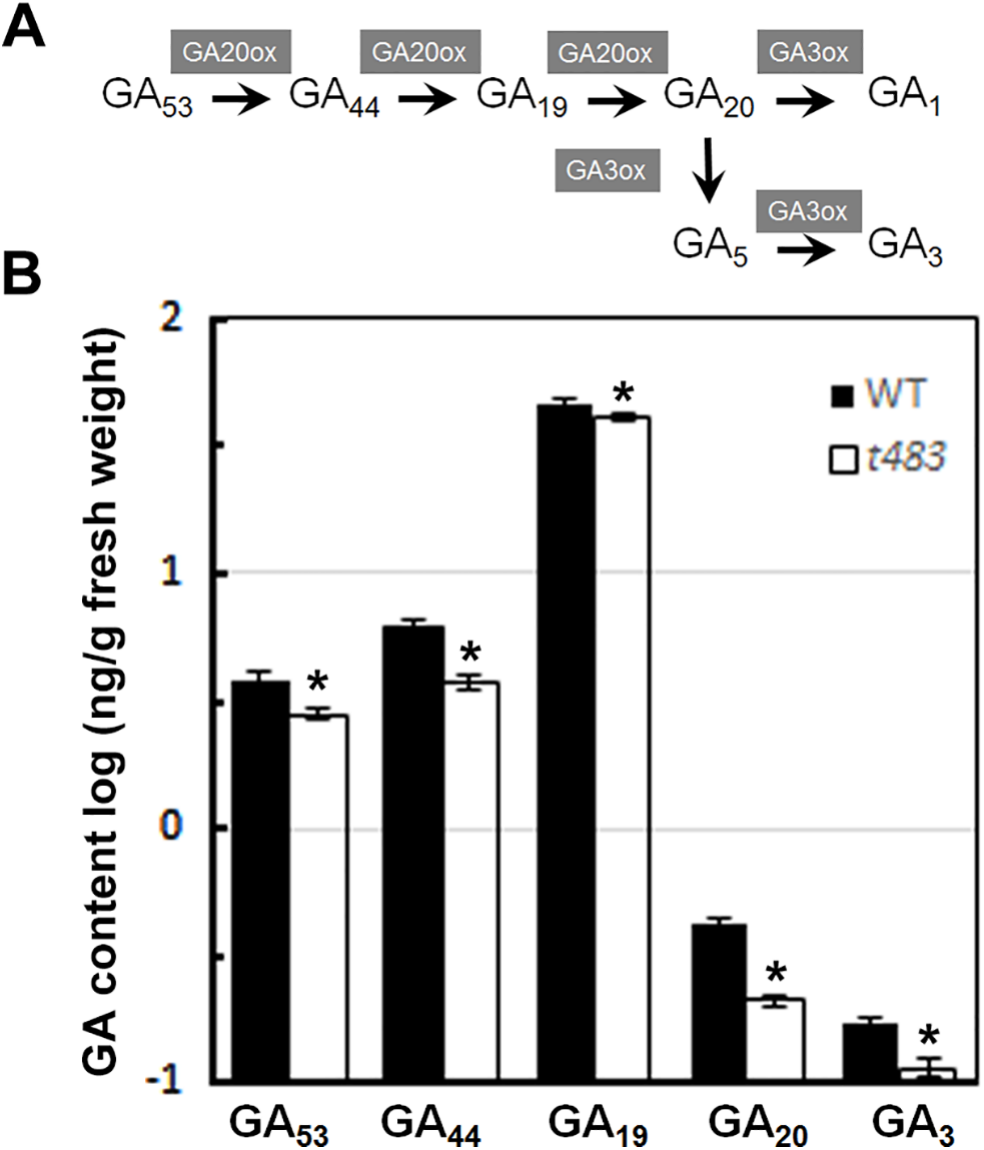
Endogenous gibberellin content in *t483* and WT. A, Schematic diagram of GA biosynthesis in the13-OH pathway. Gray boxes indicate steps catalyzed by GA20ox and GA3ox enzymes. B, Endogenous GAs levels in two-week-old *t483* and WT seedlings. GA_53,_ GA_44,_ GA_19,_ GA_20_ and GA_3_ levels are indicated by a logarithmic scale. Values are means ±SD of three samples (*P<0.05).

The *t483* mutant exhibited gibberellic acid (GA) deficiency phenotypes such as short roots, dwarfism, and late seed germination (Fig 1). In addition, the gene encoding gibberellin 20 oxidase 4 (OsGA20ox4), an active GA synthetase, was upregulated in *t483* compared to WT (S4 Table). To evaluate if GA metabolism was altered in the mutant, endogenous GA content was measured in the *t483* mutant and WT. Two weeks after germination, the levels of 13-OH GAs were significantly lower in the *t483* mutant than in WT. In particular, GA_20_ and active GA_3_ levels were reduced by 50% compared to WT (Fig 6B). These results indicate that the GA synthesis pathway is defective in *t483*.

**Fig 7:**
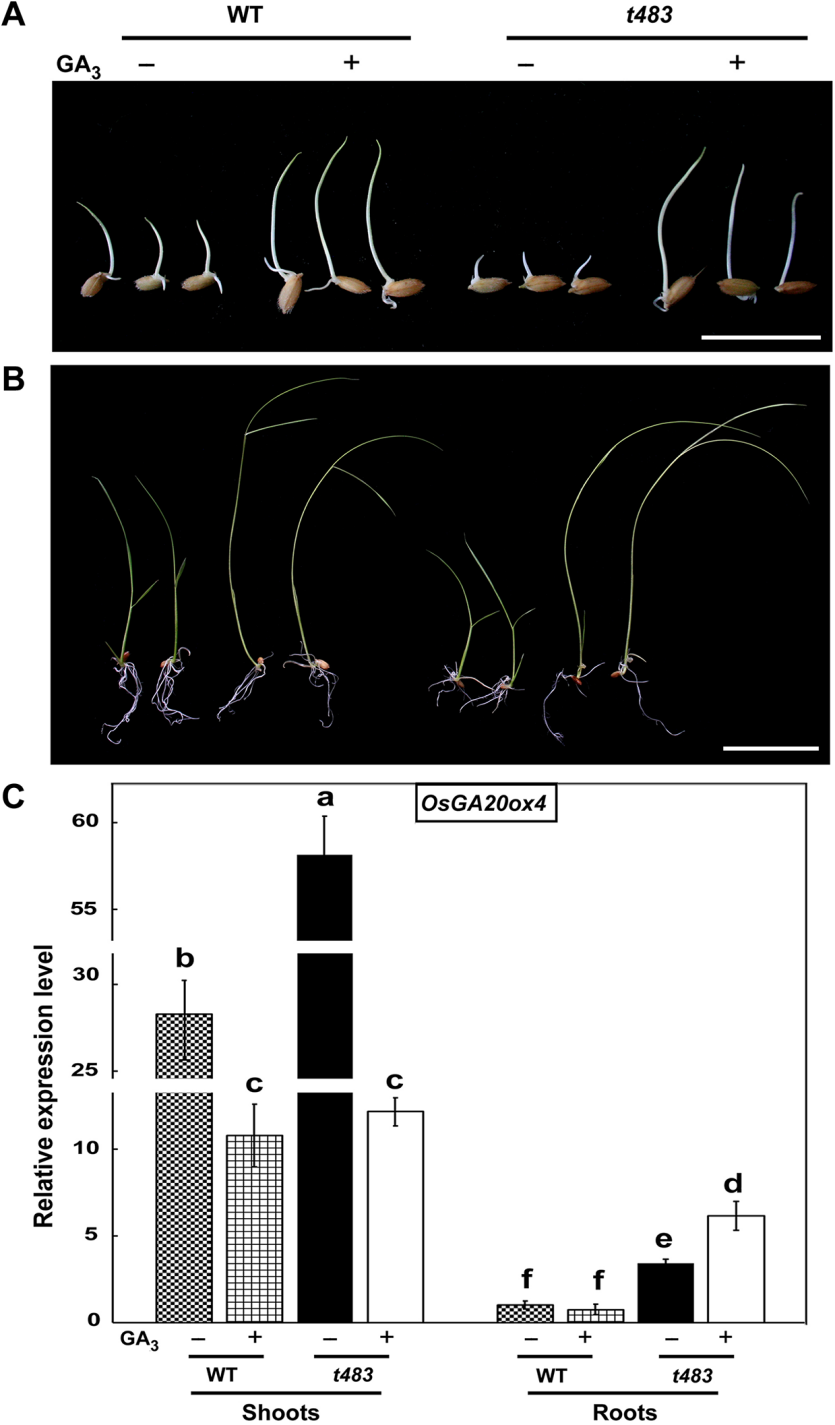
Response of *t483* to gibberellin supplementation. A, WT and *t483* seed germination with or without treatment with GA_3_ (5 μM). Image taken 3 DAI (days after imbibition). B, WT and *t483* shoot and root development with or without GA_3_ (5 μM). Image taken 14 DAI. C, Analysis of *OsGA20ox4* expression in shoots and roots of WT and *t483* seedlings with GA_3_ treatment (at 14 DAI). Values are means ±SD of three replicates. Values annotated with the same letter are not significantly different at P<0.01, with Fisher’s least significant difference test. Scale bars: 2 cm (A), 4 cm (B).

To further test the effects of GA in *t483*, WT and *t483* mutant plants were treated with exogenous GA_3_. Seed germination vigor was improved in *t483* by the addition of GA_3_ (Fig 7A), but there was no change in the overall seed germination rate (data not shown). Seedlings were also treated to investigate the ongoing response of the *t483* mutant to GA_3_. Root lengths of WT seedlings were unaffected by GA_3_ treatment, but root lengths in *t483* seedlings increased significantly to lengths similar to those in WT (Fig 7B and S1B Fig). Consistent with the morphological responses, expression of *OsGA20ox4* was more responsive to GA_3_ treatment in *t483* roots than in WT roots (Fig 7C). Shoot length was enhanced equally in WT and *t483* seedlings upon GA_3_ treatment (Fig 7B and S1C Fig). In untreated shoots, *OsGA20ox4* mRNA accumulated to a higher level in *t483* than in WT, but *OsGA20ox4* mRNA levels were decreased to similar levels in *t483* and WT when treated with GA_3_. In untreated roots, *OsGA20ox4* mRNA accumulated to higher levels in *t483* than in WT. On treatment with GA_3_, there was no change in accumulation of *OsGA20ox4* mRNA in WT, but levels increased in *t483* (Fig 7C). *CHR729* expression also responded to GA_3_ treatment (S1D Fig). These results suggest that *t483* responds to GA and that exogenous GA_3_ can overcome the mutant seed germination and root and shoot development defects, indicating that *CHR729* regulates plant development through the GA pathway.

## Discussion

A rice mutant was identified with pleiotropic phenotypic traits including dwarfism, lower tiller number, small panicle size, root growth inhibition, short and narrow leaves, and reduced chlorophyll content. The mutant gene responsible for the mutant phenotype was shown to be *CHR729*, which encoded a CHD3 protein. Our data and former reports demonstrated that mutation of this gene affected expression of a substantial number of downstream genes [16, 17, 29, 35]. The large number of DEGs identified and the pleiotropic phenotype exhibited by the mutant suggested that CHR729 might be a hierarchical regulator of transcriptional cascades in plant growth and development. RNA-seq analysis identified 500 genes that were differentially regulated in *t483* compared to WT, approximately 70% of which were upregulated in the mutant (S1 Table). About 10% of DEGs were transcription factor genes, and most of these were also upregulated in *t483* (S2 Table). The mutation in the *t483* line was predicted to lead to premature termination of CHR729. CHD3 proteins are generally considered to be transcriptional repressors, and the depression of transcription as a result of lower levels of active CHR729 is consistent with the upregulation of the majority of DEGs.

Hormones are signaling molecules that are vital to the life-cycles of many multicellular organisms [36]. There are eight major classes of plant hormones: auxin, ABA, gibberellin, ethylene, cytokinin, brassinosteroid, salicylic acid, and strigolactone. While each class performs specific functions, hormones regulate development in conjunction with one or more hormones of other classes [37, 38]. Of the DEGs identified in this study, 19 were phytohormone-related genes up- or down-regulated by CHR729 (S4 Table). These genes encoded proteins that were involved in auxin, ABA, cytokinin, gibberellin, and ethylene functions. These results suggest that CHR729 affects different plant tissues throughout development through its influence on several different phytohormone pathways.

In general, seed dormancy and germination are regulated by the antagonistic actions of ABA and GA. ABA induces and maintains the dormant seed state [39, 40], whereas GA negatively regulates dormancy by releasing coat-mediated seed dormancy and promoting or permitting seed germination [41]. Ethylene acts in concert with GA to promote seed germination, and can counteract the effects of ABA in seed germination [42]. Seed germination is thus a highly complex process that requires concerted interactions between ABA, ethylene, GA, and possibly other hormones. More research is needed to further elucidate the multiple roles of hormones in seed germination. The *t483* mutant exhibited late germination and a low rate of germination. The expression of genes related to ABA, GA, and ethylene biosynthesis was altered in the mutant, as was signaling gene expression (S4 Table). Endogenous GA levels and levels of bioactive GA_3_ were lower in the *t483* mutant than in WT (Fig 6). Addition of exogenous GA_3_ in the *t483* line rescued the late seed germination phenotype (Fig 7A), but a similar effect was not seen with added ethylene (data not shown). This suggests that the *t483* mutant is defective in GA biosynthesis and that late seed germination may be caused by the reduction in endogenous GA. As GA can regulate ethylene biosynthesis and the response to ethylene, it is possible that the differential expression of ethylene-related genes observed between WT and *t483* might also be due to low endogenous GA levels [43]. The *t483* mutant line thus constitutes a valuable genetic resource as not only can it be used to investigate the mechanisms underlying control of seed germination by phytohormones, but it can also be used to examine CHD3-mediated control of seed germination via the phytohormone pathway.

GA plays a key role in the regulation of plant growth and development, and many GA biosynthetic, catabolic, and signaling proteins have been identified [44]. While GAs are primarily known as growth-promoting hormones, they can also function as negative regulators. For example, bioactive GA in rice positively regulates seed germination and root elongation [41], but negatively regulates tillering through the *Homeobox 1* and *Teosinte branched1* pathway [45–47]. GA also negatively controls adventitious root development [48]. The *t483* mutant displayed GA deficiency phenotypes such as short roots, dwarfism, and late seed germination (Figs 1 and 2). Endogenous GA assays in WT and *t483* lines showed most of the 13-OH GAs to be present in both WT and *t483*. GA levels were lower in *t483* compared to WT, but the bioactive GA_1_ was not detected in either WT or *t483*. Although this differed from a previous report, in which GA_1_ was detected in rice seedlings in the vegetative stage [49], the conflicting results might be a consequence of different sample timings. Here, treatment with exogenous GA_3_ rescued most of the phenotypic effects seen in the mutant (Fig 7 and S1 Fig). GA deficiency generally causes thick culms; however, tiller number was reduced in *t483*. It is possible that the CHR729-controlled genes that are involved in tillering control do not act antagonistically to the GA pathway.

GA20ox is a key enzyme that catalyzes the penultimate steps in bioactive GA synthesis from GA_53_/GA_12_ to GA_20_/GA_9_. Rice contains four *GA20ox*-like genes. *GA20ox2*, also named *SD-1* (semi-dwarfing gene), is known as the rice “green revolution gene” due to the dramatic yield increases (and semi-dwarfism) seen in *sd-1* lines [50]. A second gene, *GA20ox1*, also affects plant stature, and its overexpression results in tall plants and a GA-overproduction phenotype [51]. *GA20ox3* contributes to disease resistance as well as to plant stature [52]. The function of *GA20ox4* remained unclear until now. RNA-seq and qRT-PCR analysis performed in this study showed that *GA20ox4* was upregulated in *t483* compared to WT. This result was contradictory when considered in the context of the functions of the other three *GA20ox* genes. Specifically, higher *GA20ox* levels would be expected to produce a GA-overproduction phenotype, but the *t483* mutant contained low GA levels and exhibited features characteristic of GA deficiency. This suggests that *GA20ox4* plays a different role to the other *GA20ox* genes in GA metabolism.

*CHR729* appeared to negatively control the *OsGA20ox4* gene. Compared with WT, *CHR729* expression decreased and *OsGA20ox4* increased in the *t483* mutant. GA_3_ supplementation data were consistent with these observations (Fig 7 and S1 Fig). Previous research showed that the CHD3 protein regulated target gene expression via H3K27me3 [17]. It is possible that control of *OsGA20ox4* by *CHR729* may be regulated in a similar manner. Further studies will enhance our understanding of CHR729 and its regulatory mechanisms.

## Supporting Information

S1 Fig GA_3_ treatment in WT and *t483* seedlings. A–C, Root number (A), root length (B), and shoot length (C) in WT and *t483* with or without GA_3_ treatment. Measurements were taken from 12 plants at 14 DAI. Values are means ±SD. D, Expression of *CHR729* in shoots and roots of WT and *t483* seedlings with GA_3_ treatment (at 14 DAI). Values are means ±SD of three replicates. Values annotated with the same letter are not significantly difference at P<0.01, with Fisher’s least significant difference test.
(TIF)

S1 Table Differentially expressed genes in *t483*. (XLSX)

S2 Table Transcription factor genes differentially regulated in *t483*. (XLSX)

S3 Table Genes in GO categories enriched from differentially expressed genes. (XLSX)

S4 Table Genes putatively involved in changed phytohormone responses (auxin, abscisic acid, cytokinins, gibberellin and ethylene) in *t483*. (XLSX)

S5 Table Sequences of primers used in this work. (XLSX)
