# Supplementary figures and images for: CHR729 Is a CHD3 Protein That Controls Seedling Development in Rice
Source: PLoS One. 2015 Sep 23;10(9):e0138934. doi: 10.1371/journal.pone.0138934 (PMC4580627; doi:10.1371/journal.pone.0138934)

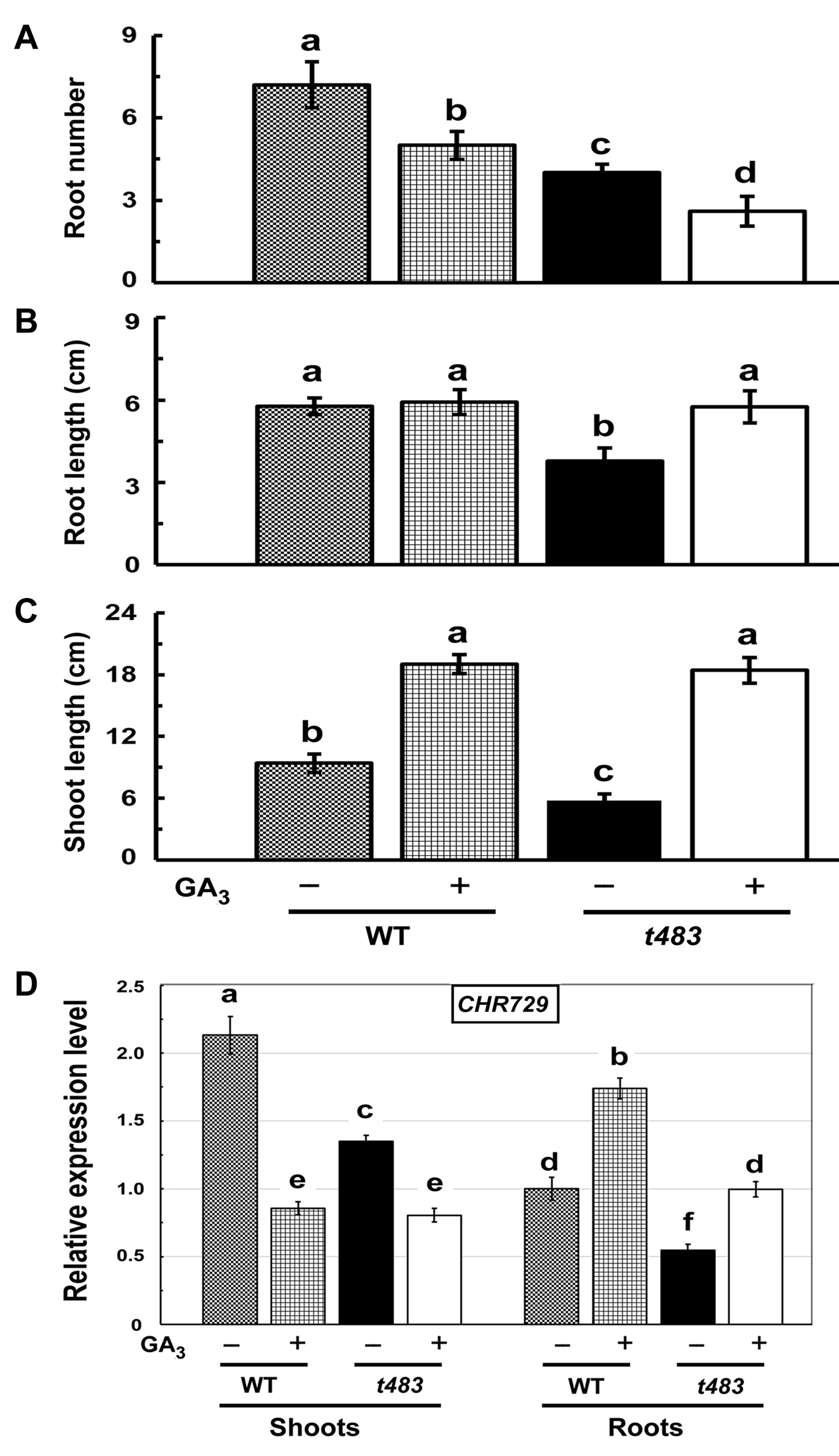

Supplement: S1 Fig — A–C, Root number (A), root length (B), and shoot length (C) in WT and t483 with or without GA3 treatment. Measurements were taken from 12 plants at 14 DAI. Values are means ±SD. D, Expression of CHR729 in shoots and roots of WT and t483 seedlings with GA3 treatment (at 14 DAI). Values are means ±SD of three replicates. Values annotated with the same letter are not significantly difference at P<0.01, with Fisher’s least significant difference test. (TIF) [file pone.0138934.s001.tif]
